# A49 TWO NOVEL LIVE BIOTHERAPEUTIC PRODUCTS PROTECT DSS-EXPOSED MICE FROM ACUTE COLITIS WHEN COMPARED TO 5-ASA

**DOI:** 10.1093/jcag/gwac036.049

**Published:** 2023-03-07

**Authors:** A Verdugo Meza, S Gill, A Godovannyi, J Barnett, N Haskey, D Gibson

**Affiliations:** Biology, University of British Columbia, Kelowna, Canada

## Abstract

**Background:**

Live biotherapeutic products (LBPs) offer a more rationalized and multitargeted approach to treating gastrointestinal diseases. BioColoniz and BioPersist are two LBPs derived from the parental strains *L. reuteri* and *E. coli* Nissle 1917 (EcN), respectively. The parental strains are known to offer some benefit in preventing relapses in IBD patients however the results are heterogeneous. To overcome this, the parental strains were approached as LBPs by introducing traits to thrive under the inflammatory conditions of the colon. Therefore, our aim is to characterize the role of these LBPs in IBD.

**Purpose:**

To evaluate the therapeutic effect of the LBPs BioColoniz and BioPersist in an acute model of colitis.

**Method:**

Female C57Bl/6 mice were treated with BioColoniz or BioPersist via oral gavage for three consecutive days prior to DSS challenge. Then mice were exposed to 3.5% DSS via drinking water for seven days. As controls, we also included mice treated with vehicle or the parental strains *L. reuteri* or EcN. In order to compare the effect of LBPs in the onset of acute colitis to current maintenance therapies for UC, we also exposed another group of mice to DSS and simultaneously administer 5-ASA. Mice were monitored daily for signs of disease and at the end of the experiment, colon tissue was collected for histopathological and molecular analysis.

**Result(s):**

The administration of BioColoniz and BioPersist delayed and decreased the colitic phenotype of mice exposed to DSS. Differences in signs of disease, such as diarrhea and weight loss, were evident by day 4 for vehicle or 5-ASA groups, whereas mice in the LBPs groups were still gaining weight. When analyzing the histopathological changes, mice in the LBPs groups presented lower scores when compared to the vehicle and 5-ASA groups. Specifically, mice treated early with BioColoniz or BioPersist presented a more preserved mucosal architecture with visible crypts. Although 5-ASA-treated mice still had vestiges of crypts, the damage in the mucosal architecture was more severe, similar to the observed in mice treated with the parental strains EcN and *L. reuteri*. We also looked at the expression of proinflammatory cytokines, finding an increase in TNFα, IFNγ, and IL-17a in mice treated with 5-ASA but not in mice treated with LBPs. However, the expression of protective factors such as mucin Muc2 or the antimicrobial peptide Reg3γ was similarly high in 5-ASA and LBP-treated mice when compared to vehicle or parental strains groups, suggesting some therapeutic commonalities between 5-ASA and our LBPs.

**Conclusion(s):**

The early administration of the LBPs BioColoniz and BioPersist protect mice from severe acute colitis, being more protective than 5-ASA. Since some differences and similarities were observed between the LBPs- and 5-ASA-treated mice, such as crypt preservation versus increased expression of some protective factors, the next step will aim to identify which mechanisms are specifically triggered by the LBPs.

**Disclosure of Interest:**

None Declared

